# Long Period Grating-Based Fiber Coupling to WGM Microresonators

**DOI:** 10.3390/mi9070366

**Published:** 2018-07-23

**Authors:** Francesco Chiavaioli, Dario Laneve, Daniele Farnesi, Mario Christian Falconi, Gualtiero Nunzi Conti, Francesco Baldini, Francesco Prudenzano

**Affiliations:** 1 Institute of Applied Physics “Nello Carrara” (IFAC), National Research Council of Italy (CNR), Via Madonna del Piano 10, Sesto Fiorentino, 50019 Firenze, Italy; d.farnesi@ifac.cnr.it (D.F.); g.nunziconti@ifac.cnr.it (G.N.C.); f.baldini@ifac.cnr.it (F.B.); 2 Department of Electrical and Information Engineering, Polytechnic University of Bari, 70125 Bari, Italy; dario.laneve@poliba.it (D.L.); mariochristian.falconi@poliba.it (M.C.F.); francesco.prudenzano@poliba.it (F.P.)

**Keywords:** microresonator, whispering gallery mode, long period grating, fiber coupling, distributed sensing, chemical/biological sensing

## Abstract

A comprehensive model for designing robust all-in-fiber microresonator-based optical sensing setups is illustrated. The investigated all-in-fiber setups allow light to selectively excite high-Q whispering gallery modes (WGMs) into optical microresonators, thanks to a pair of identical long period gratings (LPGs) written in the same optical fiber. Microspheres and microbubbles are used as microresonators and evanescently side-coupled to a thick fiber taper, with a waist diameter of about 18 µm, in between the two LPGs. The model is validated by comparing the simulated results with the experimental data. A good agreement between the simulated and experimental results is obtained. The model is general and by exploiting the refractive index and/or absorption characteristics at suitable wavelengths, the sensing of several substances or pollutants can be predicted.

## 1. Introduction

In recent years, whispering gallery mode (WGM) microresonators, such as microdisks [1], microbubbles [2] and microspheres [3], have gained much interest among researchers thanks to their capability to strongly confine the light in very compact volumes. In fact, during the several revolutions of the light signal in these resonators, the WGM field evanescently couples to the surrounding environment and even a very small change in the microresonator size and/or in the refractive index can induce significant changes in the quality factor (*Q*-factor) and/or resonance wavelengths of the microresonator. In view of this, a number of sensing applications, involving WGM microresonators, are reported in literature, such as the sensing of local temperature [2,3,4,5,6], refractive index [7], pressure [8], biological [9] and spectroscopic parameters [10]. Their huge potentiality in biosensing by means of label-free detection down to single molecules was also proved [11]. Moreover, by doping the microresonators with rare-earths, integrated light sources with narrow line emission can be obtained [12].

The combination of the peculiarities of optical fibers with WGM microresonators can provide great opportunities in the field of sensing especially. Thanks to the use of a fiber taper, high-Q WGM resonances in different types of microresonators can be efficiently excited. The taper allows for obtaining a proper evanescent electromagnetic field, which can be coupled into the microresonators, and more than 90% of the fiber mode power can be transferred on the microresonators [13]. This paves the way for several scenarios in telecommunications requiring the generation of narrow line emission. One of these is the possibility to develop an all-in-fiber coupling system for quasi-distributed and wavelength selective addressing of different WGM microresonators located along the same optical link. The first fiber-based setup for efficient coupling of light to a high-Q WGM microresonator is illustrated in [14]. It is based on a long period grating (LPG) followed by a thick fiber taper, both of which are derived from the same fiber. The LPG allows wavelength selective excitation of high-order cladding modes; in this way, thicker and more robust tapers (with waist diameters larger than 15 µm, easier to fabricate than the usual 1–2 µm tapers) can be used for coupling the cladding modes to the WGMs. However, the previous configuration does not allow interrogating more than one microresonator if the transmitted light is used to carry on the signal under investigation. The only chance could be the use of the scattered light from each microresonator, resulting in more difficult and time-consuming setup implementation. Therefore, to overcome these limitations, an improvement of the previous coupling system was recently demonstrated [15]: the system is now constituted by a pair of identical LPGs with a tapered fiber in between. The existence of the second LPG allows the light coupling back into the fiber core. Hence, all the information within the core mode is transmitted up to the end of the fiber segment and it can be collected by a single photodetector. It is important to underline that the pair of identical LPGs can operate in different wavelength bands within the range of the detector, thus allowing multiple selective coupling of spatially distributed or quasi-distributed WGM microresonators by means of different wavelengths [15].

The design of the coupling system previously mentioned requires an exhaustive model, especially for developing sensing applications [14,16]. In this work, two different sensing setups, similar to those described in [15], are considered. The two setups consist of either a microsphere or a microbubble coupled to a tapered fiber. An analytical model for simulating the two setups is detailed. The proposed approach is complete and it is well validated with the experimental results reported in literature [15].

## 2. Overview of the All-in-Fiber Coupling System

**Figure 1:**
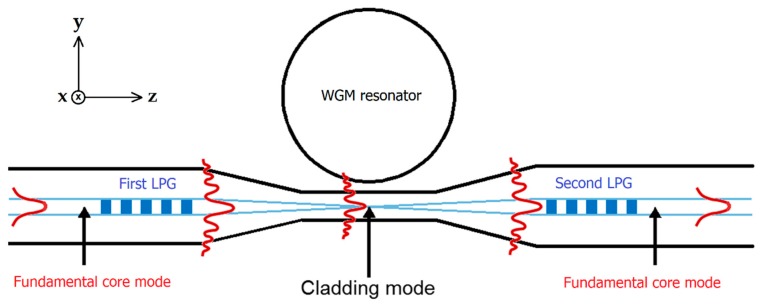
Sketch of the coupling principle of an optical whispering gallery mode (WGM) microresonator by means of a tapered fiber in between two identical long period gratings (LPGs).

Figure 1 shows a sketch of the coupling system used in this work. The system is constituted by an optical WGM microresonator coupled to a tapered fiber. On both sides of the taper, there are the two identical LPGs. The first LPG allows the coupling between the fundamental core mode and a specific cladding mode depending on the grating parameters. Then, the evanescent field of this cladding mode excites the WGMs in the microresonator. Finally, the light is coupled back from the foregoing cladding mode into the fiber fundamental mode via the second LPG.

**Figure 2:**
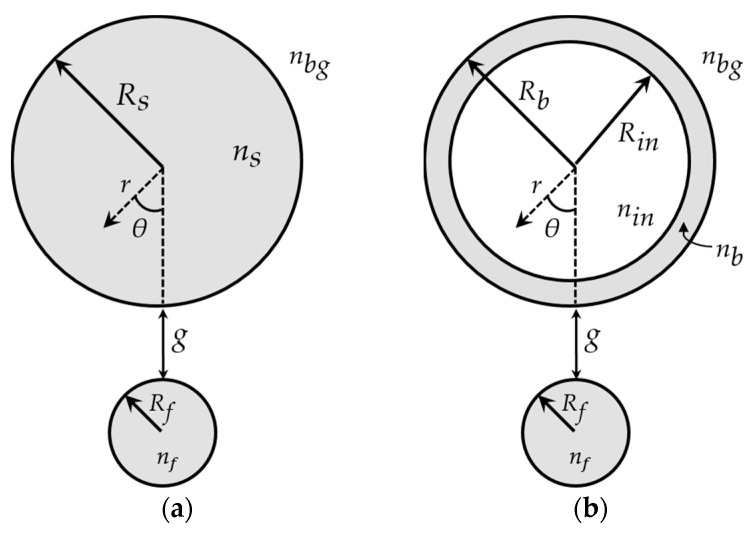
Geometrical configurations of the simulated coupling systems consisting of a tapered fiber coupled to (**a**) a microsphere and (**b**) a microbubble. The cross-section of the tapered fiber waist is shown. The light grey color represents the silica glass.

In this work, two different types of WGM microresonator are considered: microspheres and microbubbles. The cross-sections of the microresonators, together with a sketch of the tapered fiber waist, are shown in Figure 2. Figure 2a refers to the microsphere-based coupling system, whereas Figure 2b refers to the microbubble-based coupling system. The gap between each microresonator and the taper waist is *g*.

## 3. Materials and Methods

### 3.1. Manufacturing of WGM Microresonators and Optical Fiber LPGs

The model is realistic and refers to two experimental setups described in [15]. Adiabatic tapered fibers were fabricated using heating and pulling of a commercially available boron-germanium co-doped single-mode optical fiber (Fibercore PS1250/1500, Fibercore Ltd., Surrey, UK) [17]. In particular, the fiber core and cladding diameters are 6.9 μm and 124.6 μm, respectively. Tapers with a diameter among 15–18 μm are manufactured to guide/handle the cladding modes of interest. It is worth pointing out that, for an efficient coupling of these cladding modes to WGM microresonators, a partial tapering of the optical fiber is essential to shrink the optical field size and to increase the evanescent field [14]. However, the average diameter of the manufactured tapered fibers is one order of magnitude thicker than that of standard fiber tapers (1–2 μm). This allows for improving the robustness and a decreased fragility of the coupling structure in practical applications.

The coupling system depicted in Figure 1 is completed by manufacturing the pair of identical LPGs on both ends of the tapered fiber. A point-to-point technique employing a KrF excimer laser (Lambda Physic COMPex 110, Lambda Physik AG, Goettingen, Germany) is used to inscribe the gratings [18]. Two different pairs of LPGs are manufactured with a grating period, Λ, of 340 μm and 365 μm, respectively, and a grating length, *L*, of 18.7 mm and 20.1 mm, respectively (55 grating planes).

The model includes two different types of microresonators: silica microspheres and microbubbles, manufactured as in [17,19]. The diameters of these resonator range from 260 μm to 290 μm, for microspheres, and from 380 μm to 500 μm, for microbubbles. In both cases, the microresonator size is large enough, thus the free spectral range (FSR) can be considered significantly smaller than the bandwidth of the LPGs [14].

The experimental setup we used for the monitoring and registration of the transmission spectra consists of two fiber pigtailed tunable external cavity lasers (Anritsu Tunics Plus, linewidth 300 KHz, Anritsu Corporation, Kanagawa Prefecture, Japan), covering the spectral range from 1390 nm to 1640 nm and of an optical spectrum analyzer (OSA–Ando AQ6317B, Yokogawa Test & Measurement Corporation, Tokyo, Japan) for detecting the signals. The coupling mechanism is finally tested by using the same laser sources, which can be finely and continuously swept in the spectral range within few GHz, and a single photodetector connected to a commercially available oscilloscope.

### 3.2. Theoretical Analysis

In Figure 2, the cross-sections of two different kinds of optical spherical microresonators, coupled to a tapered fiber, are shown. The developed analytical model found the electromagnetic (e.m.) fields of the microresonators and the fiber by solving the Helmholtz equation in spherical and cylindrical coordinates, respectively. In particular, the e.m. fields of the microresonators are described by the well-known WGM theory [20]. Then, the coupled-mode theory allows for modeling the optical interaction between the calculated microresonator modes and fiber modes [21].

#### 3.2.1. Analytical Model of a Dielectric Microsphere

The e.m. fields of a dielectric microsphere is found by solving the following Helmholtz equation in spherical coordinates:

(1) $$\nabla^{2}\Psi \left(r,\theta ,\phi \right)+k^{2}n_{s}\Psi (r,\;\theta ,\phi )=0$$

where *k* = *ω*$\sqrt{\mu_{0}\varepsilon_{0}}$ is the wave vector in vacuum, $n_{s}$ is the refractive index of the microsphere (Figure 2a) and Ψ(r, θ, ϕ) is the electric or magnetic field component. If the polarization of the e.m. fields is supposed to be constant throughout all points in space, the solutions of Equation (1) can be expressed in the following form [20]:

(2) $$\Psi \left(r,\;\theta ,\;\phi \right){\;=\;N}_{s}\psi_{r}{(r)\psi }_{\theta }{(\theta )\psi }_{\phi }(\phi )\;$$

where $N_{s}$ is a normalization factor calculated by assuming equal to 1 the integral of ${\left|\Psi \right|}^{2}$, over all space, divided by ${2\pi R}_{s}$, $R_{s}$ is the microsphere radius (Figure 2a), and $\psi_{r}$, $\psi_{\theta }$ and $\psi_{\phi }$ are the radial, polar and azimuthal contributions of the field, respectively [20]. The separation of variables in Equation (2) allows for dividing the microsphere modes in transverse electric (TE) and transverse magnetic (TM) modes. The TE modes are characterized by having the electric field parallel to the microsphere surface, i.e., $\Psi \;\equiv E_{\theta }$, $E_{r}{=E}_{\phi }=0$. The TM modes are characterized by having the electric field perpendicular to the microsphere surface, i.e., $\Psi \;\equiv {\;H}_{\theta }$, $H_{r}{=H}_{\phi }=0$ [20]. The other field components ($H_{r}$, $H_{\phi }$ for TE modes; $E_{r}$, $E_{\phi }$ for TM modes) are determined by the boundary conditions at the interface between the microsphere and the surrounding background medium (see Figure 2a). Finally, the substitution of Equation (2) in Equation (1) gives the well-known WGM field contributions [20]:

(3) $$\;\psi_{\phi }\left(\phi \right){\;=\;e}^{\;\pm \;jm\phi }\;$$

(4) $$\psi_{\theta }\left(\theta \right)=e^{-\frac{m}{2}\theta^{2}}H_{n}\left(\sqrt{m}\theta \right),\;m\;\gg \;1\;\gg \;\theta$$

(5) $$\;\psi_{r}(r)=\{\begin{array}{c} {A\;j}_{l}\left({kn}_{s}r\right),\;r\leq R_{s}\\ {\mathrm{Be}}^{-\alpha_{s}(r\;-r_{s})},\;r>R_{s} \end{array}$$

where $H_{N}$ is the Hermite polynomial of order *N* = *l* − *m*, *l* and *m* are the mode numbers, $j_{l}$ is the spherical Bessel function of the first kind of order *l*, *A* and *B* are two constants evaluated by imposing the boundary conditions at the microsphere surface, $\alpha_{s}{=(\beta -k^{2}n_{bg}^{2})}^{1/2}$ is the exponential decay constant of the evanescent field in the background medium with refractive index $n_{bg}<n_{s}$ and $\beta_{l}$ is the propagation constant of the mode parallel to the microsphere surface [20]. The microsphere modes, WGM*_l_*_,*m*,*n*_, are uniquely described by the three integers *l*, *m* and *n*. The value *m* is the number of field maxima along the *ϕ*-direction; the value *l* − *m* + 1 is the number of field maxima along the *θ*-direction; the value *n* is the number of absolute field maxima along the *r*-direction.

By matching the tangential field components of TE and TM modes at ${r=R}_{s}$, a homogeneous linear equation system of the form Mx=0 is obtained, where ${x=[A,B]}^{T}$ and

(6) $$M=\left[\begin{array}{cc} j_{l}\left({kn}_{s}r_{s}\right) & -1\\ {kn}_{s}j_{l}^{\prime }\left({kn}_{s}R_{s}\right) & {\chi \alpha }_{s} \end{array}\right]$$

where *χ* = 1, for TE modes, and χ = $n_{s}^{2}/n_{bg}^{2}$, for TM modes. The first row of the matrix M is obtained by matching either $E_{\theta }$, for TE modes, or $H_{\theta }$, for TM modes, at ${r=R}_{s}$. The second row is obtained by matching either $H_{\phi }$, for TE modes (assuming $\partial E_{\theta }/\partial r\;\gg {\;E}_{\theta }/r$), or $E_{\phi }$, for TM modes (assuming $\partial H_{\theta }/\partial r\;\gg {\;H}_{\theta }/r$) [20]. By imposing equal to zero the determinant of the matrix M, the microsphere characteristic equation is obtained:

(7) $$\left(\frac{l}{r_{s}}+{\chi \alpha }_{s}\right)j_{l}\left({kn}_{s}R_{s}\right){=kn}_{s}j_{l+1}\left({kn}_{s}R_{s}\right)$$

where the recursion formula, $j_{l}^{\prime }\left(x\right)={lx}^{-1}j_{l}\left(x\right)-j_{l+1}(x)$, has been used [20]. Equation (7) relates the resonant wavelengths of the WGMs to the mode numbers *l* and *n*.

#### 3.2.2. Analytical Model of a Dielectric Microbubble

A microbubble can be seen as a hollow microsphere with a glass shell in which the WGMs propagate [19]. As illustrated in Figure 2b, the thickness of the glass shell is determined by the inner, $R_{in}$, and outer, $R_{b},$ radii of the microbubble. The refractive index of the glass shell is $n_{b}$, whereas the refractive index of the medium inside the microbubble is $n_{in}$. The e.m. analysis of a microbubble follows the same procedure of the previous Section 3.2.1. In this case, the radial field contribution, $\psi_{r}(r)$, takes a different form to account for the two separation interfaces:

(8) $$\psi_{r}(r)=\{\begin{array}{c} {A\;j}_{l}\left({kn}_{in}r\right),\;r\leq R_{in}\\ {Bj}_{l}\left({kn}_{b}r\right)+{C\;y}_{l}\left({kn}_{b}r\right),\;R_{in}<r\leq R_{b}\\ {\mathrm{De}}^{{-\alpha }_{s}(r-R_{b})},\;r>R_{b} \end{array}$$

where $y_{l}$ is the spherical Bessel function of the second kind of order *l*, and *A*, *B*, *C* and *D* are constants to be determined by applying the boundary conditions to the *θ*- and *ϕ*-polarized field components at ${r=r}_{in}$ and ${r=R}_{b}$. As described in the previous Section 3.2.1, the boundary conditions lead to the homogeneous linear equation system Mx=0, where ${x=[A,\;B,\;C,\;D]}^{T}$ and

(9) $$M=\left[\begin{array}{cccc} j_{l}\left({kn}_{in}R_{in}\right) & -j_{l}\left({kn}_{b}R_{in}\right) & -y_{l}\left({kn}_{b}R_{in}\right) & 0\\ n_{in}j_{l}^{\prime }\left({kn}_{in}R_{in}\right) & –\chi_{1}n_{b}j_{l}^{\prime }\left({kn}_{b}R_{in}\right) & -\chi_{1}n_{b}y_{l}^{\prime }\left({kn}_{b}R_{in}\right) & 0\\ 0 & j_{l}\left({kn}_{b}R_{b}\right) & y_{l}\left({kn}_{b}R_{b}\right) & -1\\ 0 & {kn}_{b}j_{l}^{\prime }\left({kn}_{b}R_{b}\right) & {kn}_{b}y_{l}^{\prime }\left({kn}_{b}R_{b}\right) & \chi_{2}\alpha_{s} \end{array}\right]$$

where $\chi_{1}{=\chi }_{2}=1$, for TE modes, and $\chi_{1}=n_{in}^{2}/n_{b}^{2}$, $\chi_{2}=n_{b}^{2}/n_{bg}^{2}$, for TM modes. The first two rows of the matrix M are obtained by matching either $E_{\theta }$, $H_{\phi }$, for TE modes, or $H_{\theta }$, $E_{\phi }$, for TM modes, at $r=R_{in}$. The third and fourth rows are obtained by matching the same tangential fields at $r=R_{b}$. The assumptions $\partial E_{\theta }/\partial r\;\gg {\;E}_{\theta }/r$ and $\partial H_{\theta }/\partial r\;\gg {\;H}_{\theta }/r$ hold in this case too. By imposing equal to zero the determinant of the matrix M in Equation (9), the characteristic equation for the microbubble is obtained.

#### 3.2.3. Coupling Model

The coupled mode theory (CMT) [21] is applied to model the optical coupling between the microresonator ${\mathrm{WGM}}_{l,m,n}$ modes and the tapered fiber ${\mathrm{LP}}_{0,X}$ cladding modes, where the 0 and *X* subscripts represent the azimuthal and radial orders, respectively, of the linearly-polarized (LP) cladding modes. The analytical model takes into account the coupling of modes both in space and in time formulations [20,21].

The optical interaction between the fiber field, $F_{X}$, and the microresonator field, $\Psi_{l,m,n}$, is obtained and calculated from the following overlap integral [20]:

(10) $$\kappa_{xy}(z)=\frac{k^{2}}{{2\beta }_{f}}(n_{eff}^{2}-n_{bg}^{2})\iint \Psi_{l,m,n}\;\cdot F_{X}^{*}\;dxdy$$

where $\beta_{f}$ is the propagation constant of the LP mode and $n_{eff}$ is the effective refractive index of the microresonator, which is related to the propagation constant of the WGM [20]. The integration in Equation (10) is carried out over the transverse *xy*-plane at a fixed point along the tapered fiber, whose longitudinal axis is directed along the *z*-axis (see Figure 1). Then, $\kappa_{xy}(z)$ is integrated along the *z*-axis over the interaction length, *L*, i.e., κ = $\int_{L}\kappa_{xy}(z)dz$. κ is the power coupling constant, whereas $\kappa^{2}$ is the fraction of the power transferred from the fiber to the microresonator over the interaction region [21]. It should be noted that $\kappa_{xy}$ is proportional to $e^{-j\Delta \beta z}$, where ${\Delta \beta =\beta }_{f}-\beta_{m}$ is the phase mismatch between the LP mode and the WGM, whose propagation constant is either $\beta_{m}={m/R}_{s}$, for a microsphere, or $\beta_{m}={m/R}_{b}$, for a microbubble [20].

The power coupling constants κ is also related to the time evolution of the coupled modes. By considering the microresonator as a lumped oscillator of energy amplitude $a_{\mathrm{WGM}}(t)$, the (weak) power coupling with the fiber induces a (slow) time variation of $a_{\mathrm{WGM}}(t)$, which can be expressed by means of the following rate equation [21]:

(11) $$\frac{da_{\mathrm{WGM}}(t)}{dt}=\left({j\omega }_{\mathrm{WGM}}-\frac{1}{\tau_{0}}-\frac{2}{\tau_{e}}\right)a_{\mathrm{WGM}}\left(t\right)-j\sqrt{\frac{2}{\tau_{e}\tau_{r}}}a_{in}(t)$$

where $\tau_{0}{=Q}_{0}{/\omega }_{\mathrm{WGM}}$ is the amplitude decay time-constant due to the intrinsic loss phenomena of the microresonator (including surface scattering and absorption and curvature losses), $Q_{0}$ is the intrinsic quality factor, $\omega_{\mathrm{WGM}}$ is the ${\mathrm{WGM}}_{l,m,n}$ resonant frequency, $\tau_{e}{=Q}_{e}/\omega$ is the decay time-constant related to the coupling with the fiber, $Q_{e}{=m\pi /\kappa }^{2}$ is the external quality factor, κ is the foregoing power coupling constant, ω is the input excitation frequency, $\tau_{r}{=2\pi R/v}_{g}≅{2\pi Rn}_{eff}/c$ is the revolution time of either the microsphere (${R=R}_{s}$) or the microbubble (${R=R}_{b}$) and $a_{in}(t)$ is the energy amplitude of the excitation signal at the taper input section [20,21].

The transfer characteristic of the coupling system is found by considering the steady state form of the Equation (11), thus obtaining $a_{\mathrm{WGM}}$, and then applying the following power conservation rule [21]:

(12) $$\;\frac{{\left|a_{in}\right|}^{2}}{\tau_{r}}=\frac{1}{\tau_{r}}{\left|a_{in}\;-\;j\sqrt{\frac{{2\tau }_{r}}{\tau_{e}}}a_{\mathrm{WGM}}\right|}^{2}+\frac{2}{\tau_{e}}{\left|a_{\mathrm{WGM}}\right|}^{2}$$

where the first term on the right-hand side is the non-resonant power transmitted directly to the fiber output section, while the second term is the resonant power coupled out of the microresonator [21]. Therefore, the transmittance of the system can be expressed as:

(13) $$T=\frac{{\left|a_{out}\right|}^{2}}{{\left|a_{in}\right|}^{2}}$$

where $a_{out}$ is the amplitude of the signal at the fiber output section. i.e., ${\left|a_{out}\right|}^{2}/\tau_{r}$ is equivalent to the right-hand side of Equation (12).

In the foregoing analytical model, effects of the LPGs in the transmittance calculation can be neglected, since the LPGs simply allow the selective fiber mode excitation. The calculation of the mode excitation strength is not significant and can be avoided since in the transmittance calculation the output light is normalized with respect to the input one (see Equation (13)).

## 4. Results and Discussion

This section consists of two parts: Section 4.1 presents the numerical results achieved by using the theoretical analysis described in Section 3.2, whereas Section 4.2 shows some experimental results related to possible distributed sensing with WGM microresonators.

### 4.1. Numerical Results

The e.m. fields and the propagation constants of the microresonator WGMs and the fiber LP modes simulated with the analytical model were successfully validated via a finite element method (FEM) commercial code. Moreover, the results simulated with the overall developed analytical model have been validated with the experiment reported in [15]. In the following, the simulated results are obtained via the analytical model.

The simulations are carried out by employing the experimental parameters detailed in Section 3.1. In particular, an adiabatic fiber taper with a radius $R_{f}=9\;\mu m$ is considered. The simulated microsphere and microbubble are made of silica glass. The dispersion effect on the refractive index of silica is taken into account via a proper Sellmeier formula [22]. The microsphere radius is $R_{s}=145\;\mu m$, whereas the microbubble external and internal radii are $R_{b}=200\;\mu m$ and $R_{in}=196.7\;\mu m$, respectively. The simulations are performed by considering air as the surrounding background medium. Moreover, the microbubble is considered empty. According to [15], the simulated wavelength range is centered on $\lambda_{c}$ = 1613.3 nm.

To find the optimal value of the gap *g*, a number of simulations are performed by considering different gap values, *g* = 0, 10, 100, 200, 500, 1000 nm. For each value of *g*, the simulated transmittance *T* is compared with the experimental one. Both setups employing the microsphere and the microbubble are taken into account.

**Figure 3:**
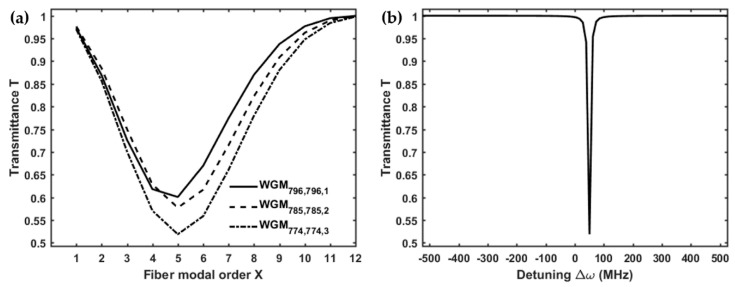
(**a**) Transmitted power of the microsphere-based setup, calculated for three resonant ${\mathrm{WGM}}_{l,m,n}$, as a function of the modal order *X* of the ${\mathrm{LP}}_{0,X}$ cladding modes, with a gap *g* = 0 nm; (**b**) transmission of the ${\mathrm{WGM}}_{774,774,3}$, excited by the ${\mathrm{LP}}_{0,5}$, as a function of the detuning Δω, with a gap *g* = 0 nm; microsphere-based setup.

Figure 3a shows the transmittance *T* of the microsphere-based setup, calculated for three different ${\mathrm{WGM}}_{l,m,n}$, as a function of the radial order *X* of the ${\mathrm{LP}}_{0,X}$ cladding modes, considering a gap *g* = 0 nm (i.e., taper and microsphere in mechanical contact, as in [15]). The lowest transmittance dip, which corresponds to the highest coupling with the microsphere, can be attained for the ${\mathrm{WGM}}_{774,774,3}$ by exciting with the fiber ${\mathrm{LP}}_{0,5}$ cladding mode through the LPG. The resonance of the ${\mathrm{WGM}}_{774,774,3}$, expressed in terms of the detuning Δω, is shown in Figure 3b. The simulated results are in good agreement with the experiment reported in [15]. In fact, by exciting the ${\mathrm{WGM}}_{774,774,3}$, the simulated transmittance of the microsphere-based coupling system reaches a minimum of *T* = 0.52, while, in the experiment, the measured transmittance is about *T* = 0.65. The small discrepancy can be explained by considering that the actual total losses are higher. However, it is worth nothing that, in Figure 3a, the transmittance simulated for the ${\mathrm{WGM}}_{774,774,3}$ coupled with the ${\mathrm{LP}}_{0,7}$ is *T* = 0.66, practically coincident with the measured transmittance [15], for the same fiber modal order.

**Figure 4:**
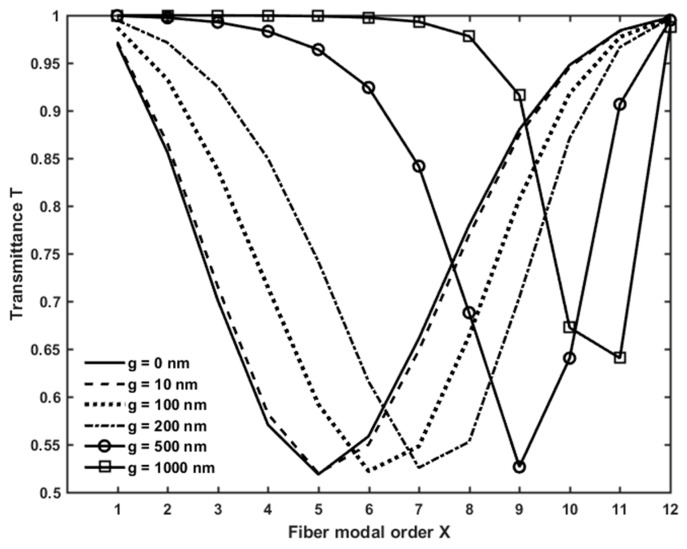
Transmitted power of the microsphere-based setup, calculated for different gap values, ranging from g = 0 nm to g = 1000 nm.

Figure 4 shows the transmittance *T* of the microsphere-based setup, calculated for the gap values, *g* = 0, 10, 100, 200, 500, 1000 nm. For each value of *g*, the transmittance due to the ${\mathrm{WGM}}_{774,774,3}$, which exhibits the lowest dip among the coupled WGMs, is plotted as a function of the radial order *X* of the fiber modes. In other words, the ${\mathrm{WGM}}_{774,774,3}$ transmittance is predominant with respect to the contribution of the other WGMs. Except for large gap values (*g* = 1000 nm), the simulated minimum transmittance is almost the same in all cases. Instead, the radial order of the fiber modes slightly increases as the gap increases, revealing the influence of *g* on the phase matching between the WGMs and LP modes.

**Figure 5:**
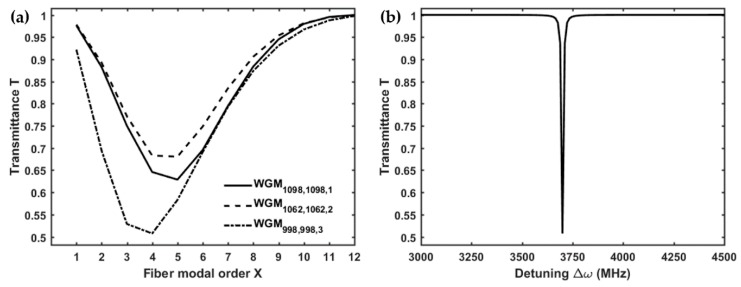
(**a**) Transmitted power of the microbubble-based setup, calculated for three resonant ${\mathrm{WGM}}_{l,m,n}$, as a function of the modal order X of the ${\mathrm{LP}}_{0,X}$ cladding modes, with a gap g = 0 nm; (**b**) transmission of the ${\mathrm{WGM}}_{998,998,3}$, excited by the ${\mathrm{LP}}_{0,4}$, as a function of the detuning Δω, with a gap g = 0 nm; microbubble-based setup.

Figure 5a reports on the transmittance *T* of the microbubble-based setup, calculated for three different ${\mathrm{WGM}}_{l,m,n}$, as a function of the radial order *X* of the ${\mathrm{LP}}_{0,X}$ cladding modes, considering a gap *g* = 0 nm (i.e., taper and microbubble in mechanical contact, as in [15]). The lowest transmittance dip, which corresponds to the highest coupling with the microbubble, can be attained for the ${\mathrm{WGM}}_{998,998,3}$ by exciting with the fiber ${\mathrm{LP}}_{0,4}$ cladding mode through the LPG. The resonant detuning of the ${\mathrm{WGM}}_{998,998,3}$, corresponding to the wavelength λ = 1618.4 nm, is shown in Figure 5b. The simulated transmittance of the microbubble-based coupling system reaches a minimum of *T* = 0.51, practically coincident with the measured value [15] even if referring to a slightly lower fiber modal order.

**Figure 6:**
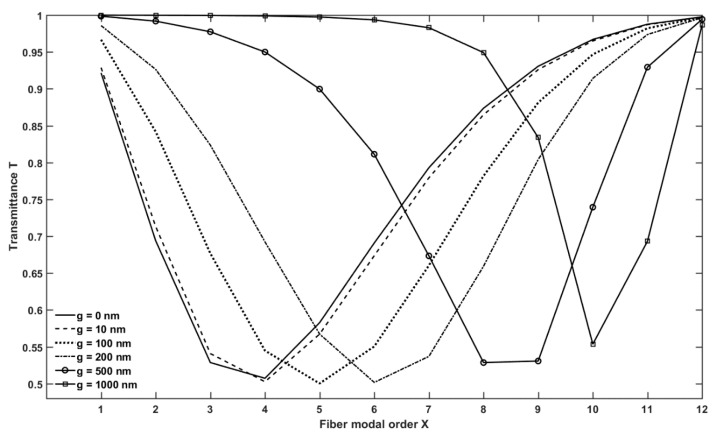
Transmitted power of the microbubble-based setup, calculated for different gap values, ranging from g = 0 nm to g = 1000 nm.

Figure 6 shows the transmittance *T* of the microbubble-based setup, calculated for the gap values, *g* = 0, 10, 100, 200, 500, 1000 nm. For each value of *g*, the transmittance due to the ${\mathrm{WGM}}_{998,998,3}$, which exhibits the lowest dip among the coupled WGMs, is plotted as a function of the radial order *X* of the fiber modes. In this case, the ${\mathrm{WGM}}_{998,998,3}$ transmittance is predominant with respect to the contribution of the other WGMs. As in the microsphere case, except for large gap values (*g* = 1000 nm), the simulated minimum transmittance is almost the same in all cases, while the radial order of the fiber modes slightly increases as the gap increases. It is worthwhile noting that, in Figure 4 and Figure 6, the critical coupling condition can be achieved by considering different fiber optic modal order.

**Figure 7:**
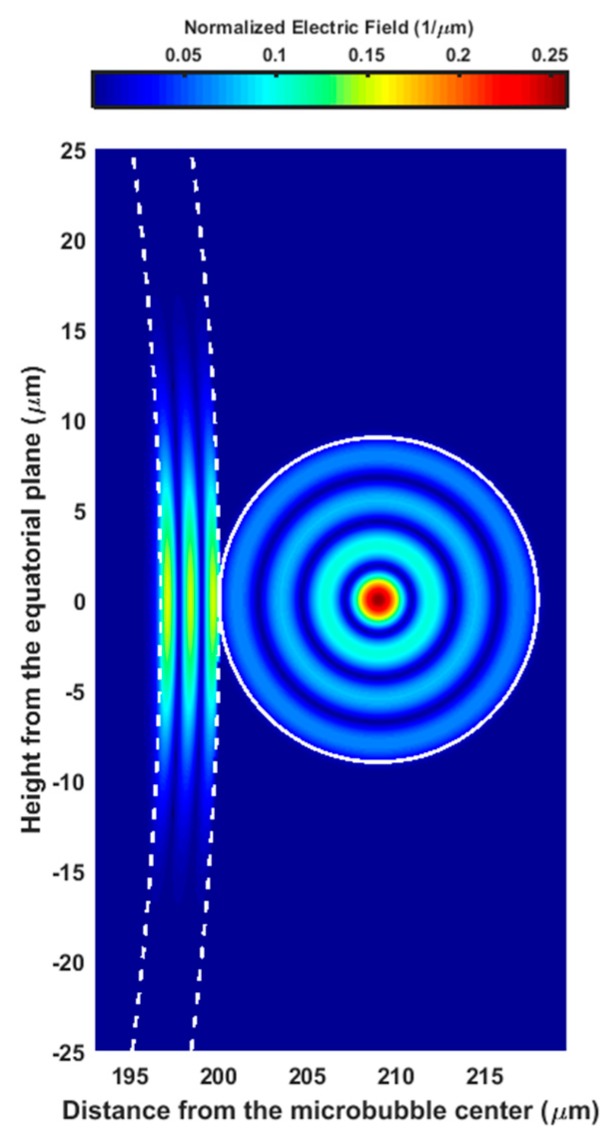
Distribution of the normalized electric fields confined in the microbubble glass layer (on the left) and in the fiber taper (on the right) for the ${\mathrm{WGM}}_{998,998,3}$ coupled with the ${\mathrm{LP}}_{0,4}$, gap g = 0 nm.

Figure 7 illustrates the normalized electric field of the microbubble ${\mathrm{WGM}}_{998,998,3}$ evanescently coupled to the normalized electric field of the fiber ${\mathrm{LP}}_{0,4}$ cladding mode. The four radial maxima of the ${\mathrm{LP}}_{0,4}$ is evident as well as the third radial order of ${\mathrm{WGM}}_{998,998,3}$.

### 4.2. Experimental Results

Towards the development of an all-in-fiber distributed sensing system, the coupling system illustrated in Figure 1 has been tested and high-Q WGM resonances in both microspheres and microbubbles have been proved to be effectively excited [15]. The mechanical contact between the microresonators and the tapered fiber is provided in order to avoid any environmental perturbation. Moreover, the phase-matching conditions between the fiber cladding modes and the WGMs are satisfied due to the azimuthal and radial high-order modes of the spherical microresonators [14]. The transmission dips has been fitted by a Lorentzian function obtaining typical *Q*-factor values ranging from 10^6^ up to 10^8^, for both types of microresonators, with a maximum coupling efficiency (or resonance contrast) of about 50%–60%.

**Figure 8:**
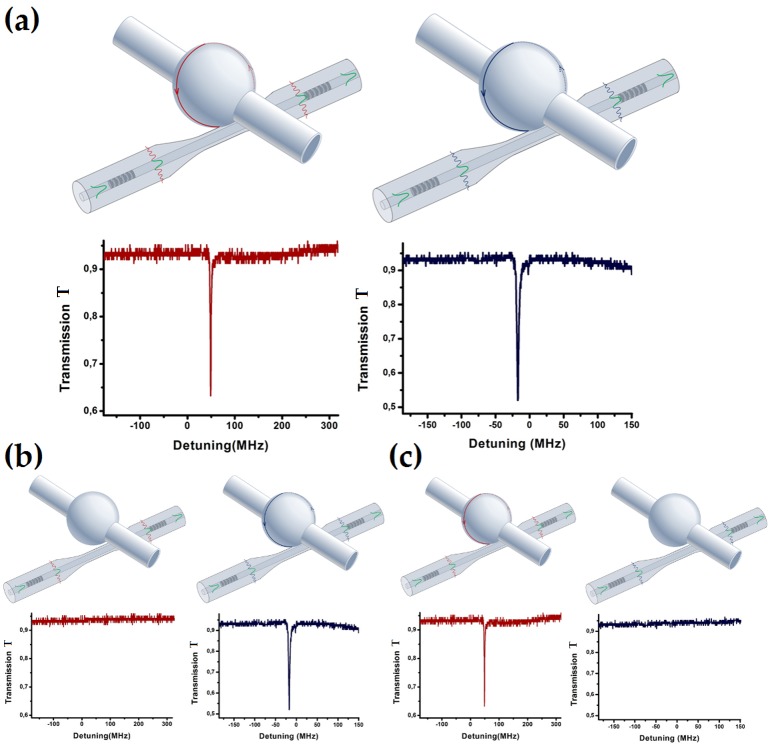
(**a**) Sketch and results of two in-series coupling systems with both the microresonators (microspheres in this case) coupled to each tapered fiber. The corresponding resonances achieved by scanning a laser source around the resonant wavelengths of the LPGs (0 MHz detuning) are also detailed below each sketch. The other two cases are shown to prove the zero cross-talk of the approach proposed; (**b**) the second microsphere is in contact, while the first is not; (**c**) the first microsphere is in contact, while the second is not.

Afterwards, the coupling system in Figure 1 is doubled along the same fiber link as a proof-of-concept test. In fact, by a proper design of the LPGs, the coupling system in Figure 1 can be replicated as many times as the effective bandwidth of both source and detector allows. A total bandwidth allocation not less than 40 nm for each pair of LPGs should be taken into account [15]. Figure 8a accounts for the resonances achieved by scanning about 2 GHz around the LPG central wavelengths (1518.9 nm for the first coupling unit and 1613.3 nm for the second one), when the microresonators of the two coupling units are in mechanical contact with their respective fiber tapers (as sketched on the top of Figure 8a). The *Q*-factor values are comparable to those obtained with the analytical model. In order to prove that the microresonators of the two coupling units can be independently excited without cross-talk, a selectivity test has been performed. By alternatively de-coupling one of the microresonators and by looking at the transmission spectrum of the other one, it is possible to prove the selective excitation, as detailed in Figure 8b (the second microresonator is in contact, while the first is not in contact) and in Figure 8c (the first microresonator in contact, while the second not in contact). As further proof and evidence of this, additional measurements performed by varying the coupling position of the tapered fiber along the azimuthal axis of the microresonators confirm our findings. Therefore, the proposed all-in-fiber coupling system can be effectively used for distributed sensing.

## 5. Conclusions

A complete model for designing an all-in-fiber coupling system allowing the wavelength selective excitation of spatially distributed or quasi-distributed optical WGM microresonators has been developed. The microresonators are evanescently side-coupled with a fiber taper to increase the coupling efficiency. A pair of identical LPGs, with the microresonator and the tapered fiber in between, can be used to excite the WGMs by means of peculiar cladding modes. The pair of identical LPGs can operate in different wavelength bands allowing multiple selective interrogation of several microresonators along the same optical fiber. The model has been validated with experimental data by considering microsphere- and microbubble-based setups. The simulated results suggest that the coupling system can be effectively used for distributed sensing applications of chemical/biological fluids. The theoretical and experimental analysis and results could open up novel opportunities in the field of chemical/biological sensing [23]. In particular, the all-in-fiber coupling system could bring about a very promising optical platform for multiplexing hollow WGM microstructures, such as microbubble-based resonators, for which an integrated microfluidics is perfectly fitted.
